# An update on long intergenic noncoding RNA p21: a regulatory molecule with various significant functions in cancer

**DOI:** 10.1186/s13578-020-00445-9

**Published:** 2020-06-22

**Authors:** Roya Amirinejad, Mina Rezaei, Zeinab Shirvani-Farsani

**Affiliations:** 1 Genetics Department, Breast Cancer Research Center, Motamed Center Institute, ACECR, Tehran, Iran; 2 Department of Cell and Molecular Biology, Faculty of Life Sciences and Biotechnology, Shahid Beheshti University G.C, Tehran, Iran

**Keywords:** *lincRNA*-*p21*, Biomarker, miRNAs, Metastasis, Angiogenesis, Apoptosis

## Abstract

Long intergenic noncoding RNA p21 was mapped on the human chromosome 6p21.2. Accordingly, it was firstly described by promoting the p53-dependent apoptosis in the mouse. Also, it is a new lncRNA playing some vital roles in the cell cycle, apoptosis, cell proliferation, tumorigenesis, invasion, metastasis, and angiogenesis. In this regard, it was shown that, *lincRNA*-*p21* regulates these biological processes involved in carcinogenesis through various signaling pathways including Notch signaling, JAK2/STAT3, and AKT/mTOR pathways. Another mechanism by that *lincRNA*-*p21* can affect these processes is a cross-talk with different miRNAs. In vitro and in vivo studies revealed dysregulation of *lincRNA*-*p21* in various human cancers. In addition, emerging evidence demonstrated that, *lincRNA*-*p21* can be considered as a potential prognostic and therapeutic biomarker in cancers. Also, *lincRNA*-*p21* enhances the response to radiotherapy for colorectal cancer. However, the molecular mechanisms of *lincRNA*-*p21* in carcinogenesis have not been fully elucidated so far. So, this review summarizes the function of *lincRNA*-*p21,* as a tumor suppressor factor in different biological processes implicated in cancers.

## Background

*LincRNA*-*p21* (Long intergenic noncoding RNA p21,), which was mapped on human the chromosome 6p21.2, upstream of the cell-cycle regulator gene p21/Cdkn1a, was firstly described as an inducer of the p53-dependent apoptosis in mouse embryonic fibroblasts [1]. *LincRNA*-*p21* has two isoforms that both of them contain a single exon and Alu inverted repeat elements. Also, *LincRNA*-*p21* together with lncRNA NEAT1 localizes in HCT-116, MCF-7, and U2OS cells [2]. *LincRNA*-*p21*, also called as TRP53COR1 (P53 Pathway Corepressor 1 protein tumor), is a new lncRNA that has an important function in initiating and progressing various cancers. Moreover, it was shown that, *lincRNA*-*p21* induces the transcriptional activity of wild-type p53 on vascular smooth muscle cells [3]. Also, it suppresses expression through either targeting mRNAs or recruiting protein binding partners to the chromatin location [4], because it contains several motifs interacting with the mRNA targets, different miRNAs, and RNA-binding proteins [5]. In addition, multiple evidence has reported that, *lincRNA*-*p21* involves in the development of different tumors. Accordingly, the lincRNA-p21 overexpression can suppress tumor invasion by Notch pathway [6]. Also, *lincRNA*-*p21* suppresses the progression of prostate cancer by apoptotic induction and controlling gene expressions downstream of p53 [7]. In fact, it plays a significant role as a moderator in different processes such as DNA damage response, apoptosis, and in cell proliferation [8]. However, the mechanisms and functions of *lincRNA*-*p21* are still unknown in the progression and development of various cancers. Likewise, *lincRNA*-*p21* are induced by DNA damage response. Then, *lincRNA*-*p21* recruit hnRNP-K (heterogeneous nuclear ribonucleoprotein K) on the promoter of p21, which is crucial for the interaction between the p53 and the p21 promoters and eventually in the enhancement of p21 expression [4]. In vivo and in vitro studies of cancer have indicated the tumor suppressor functions of *lincRNA*-*p21* in different cancer types [9–11]. Several studies have found that, *lincRNA*-*p21* has significantly reduced in multiple cancers such as gastric cancer [12], chronic lymphocytic leukemia [13], hepatocellular carcinoma [6], non-small cell lung cancer [14], colorectal cancer [15], diffuse large B cell lymphoma [16], and prostate cancer [7]. Besides presenting regulatory function, *lincRNA*-*p21* was detectable in body fluids such as blood, serum, plasma, and urine. Therefore, it can be suggested as a potential biomarker for prostate cancer [17]. Overall, few studies have been conducted on various cancers, the results of which are summarized in Table 1. In this review, we discussed the functions of lincRNA-p21 in various biological processes including tumorigenesis, and then described further evidence for the effects of lincRNA-p21 on the signaling pathways as well as a cross-talk between lincRNA-p21 and miRNAs involved in cancers.

**Table 1:** The pattern *of lincRNA*-*p21* expression and its roles in various cancers

Cancer type	Sample type		Expression pattern	Gene interplay (Direct or Indirect)	Signaling pathways	Reference
	Cell line	Patients				
Hepatocellular carcinoma	HepG2 and SMMC-7721	HCC tumor tissue	Down-regulated	Hes-1, NICD, E-cadherin, and Claudin-1	Notch and EMT signal pathways	[6]
Prostate cancer	LNCaP, DU145, PC3, PTN2 and BPH-1	Prostatectomy specimens	Down-regulated	p53, Mdm2, Puma, Noxa and Bax	P53 Signaling	[7]
Head and neck squamous cell carcinoma	HN4, HN6, HN30 HEK293T, Cal27, SCC25, Detroit562, MCF7, and MDA-MB-231	HNSCC tumor tissues	Down-regulated	p53 and NF-YA	JAK2/STAT3 signaling	[9]
Skin cancer	Balb/MK2 keratinocytes, NHEKs	–	Down-regulated	P53	p53 pathway	[10]
Gastric cancer	MGC-803, MKN-45, BGC-823, MKN-28, SGC-7901 and normal GES-1	GC tissues	Down-regulated	imentin and N-cadherin, YAP, β-catenin and NF-κB, P-ERK	Hippo pathway	[12]
Chronic lymphocytic leukemia	LY-47, BL-60 and BL-7, BJAB, BL-2, and CA-46	PBMC from patients	Up-regulated after DNA damage	P53, p21	p53 pathway	[13]
Non-small cell lung cancer	H23, H1299, and HCC-44	NSCL tumor tissue	Down-regulated	Secreted vascular endothelial growth factor A (VEGFA)	–	[14]
Colorectal cancer	CRC cell lines HCT-116 (p53þ/þ) and HCT-116 (p53−/−)	CRC specimens	Down-regulated	P53	–	[15]
B cell lymphoma	–	DLBCL tissues	Down-regulated	Cyclin D1, CDK4 and p21	–	[16]

EMT: Epithelial–mesenchymal transition; DLBCL: diffuse large B cell lymphoma; CRC: colorectal cancer; NSCL: non-small cell lung cancer; PBMC: peripheral blood mononuclear cells

### *LincRNA*-*p21* in apoptosis, cell proliferation, and cell cycle

**Fig. 1:**
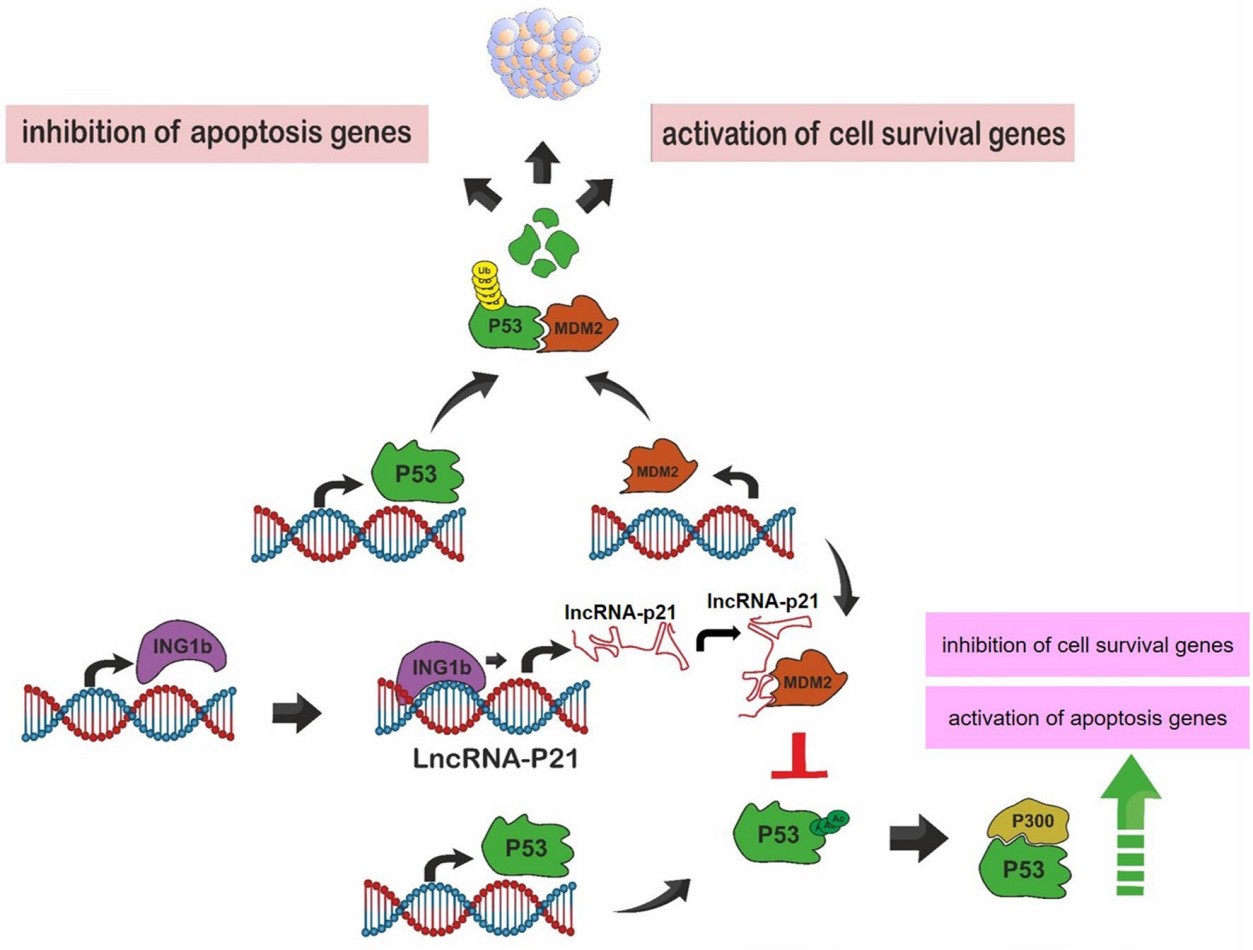
LincRNA-p21-induced apoptosis via ING1B and p53. MDM2 monoubiquitinates p53, which leads to its degradation by the proteasome complex. Consequently, the apoptosis genes are inhibited and cell survival genes are activated, resulting in the inhibition of apoptosis (up). On the other hand, ING1b leads to the overexpression of lincRNA-p21. Then, *lincRNA*-*p21* binds to MDM2 and detaches it from p53, which results in the increased p53 stability, the induced apoptosis genes, and the suppressed survival cell genes (down)

Recent studies have indicated that, *lincRNA*-*p21* is a significant regulator of apoptosis and cell proliferation by p53 signaling and inhibiting the target gene translation. For instance, Huarte et al. [1] found that, *lincRNA*-*p21* with p53 can control the number of apoptosis and cell proliferation regulator genes. They also observed that, *lincRNA*-*p21* and p53 knockdowns lead to the inhibition of the apoptosis genes such as Perp and Noxa, as well as the activation of cell survival genes such as Stat3, Atf2, and Bcl2l3. *Also, lincRNA*-*p21* overexpression results in cellular apoptosis after the induction of DNA damage [1]. Moreover, Wu et al. [3] have reported that, *lincRNA*-*p21* interacts with MDM2, thus releasing p53 from MDM2, and p53 binds to p300, resulting in the increase of p53 activity [3]. Moreover, another study demonstrated that, the apoptosis induction by ING1b is dependent on the *lincRNA*-*p21* expression [18]. Accordingly, this impact has significantly increased by p53. Furthermore, ING1b can bind to the *lincRNA*-*p21* promoter and also controls the level of *lincRNA*-*p21*. In addition, it is necessary for inducing *lincRNA*-*p21* by p53 [18]. Therefore, *lincRNA*-*p21* may cross-talk to p53 and ING1b signaling pathways (Fig. 1). Hall et al. [10] have also reported that, the down-regulation of *lincRNA*-*p21* suppressed UVB-induced apoptosis in keratinocytes from the human and mouse, while this reduced *lincRNA*-*p21* expression had no impact on the cell proliferation in keratinocytes before and after the treatment with UVB. Also, a mutation in a single p53 allele blocks the activation of UVB-induced expression of *lincRNA*-*p21,* and then inhibits the apoptosis. Accordingly, this allele may be a pro-oncogenic allele in skin cancer [10]. On the contrary, Yang et al. [19] have observed that, *lincRNA*-*p21* was considerably enhanced in the tissues of human non-small cell lung cancer and cells thereby significantly suppressed the cell apoptosis. In other words, the overexpression of *lincRNA*-*p21* repressed apoptosis, while its knockdown increased the apoptosis. The impact of *lincRNA*-*p21* on apoptosis was mediated by downregulation of the PUMA (p53 upregulated modulator of apoptosis) [19]. PUMA is a target of p53 by having significant functions in the regulation of apoptosis [20]. In fact, PUMA is a pro-apoptotic factor that represses some anti-apoptotic factors including Mcl-1, Bcl-2, Bcl-XL, Bcl-W, and A1 and also activates apoptosis [21].

The results of another investigation have demonstrated that, down-regulation of *lincRNA*-*p21* enhanced the percentage of the cells in S phase and decreased the apoptosis. Whereas, up-regulation of *lincRNA*-*p21* resulted in G1 arrest in HN6 and Cal27 cells as well as reducing the expression levels of several cell cycle regulating factors such as Cyclin B1 and Cyclin D1 along with the apoptosis induction in head and neck squamous cell carcinoma (HNSCC) cells. Collectively, *lincRNA*-*p21* suppressed the HNSCC cell growth, activated cell apoptosis, and induced G1 phase arrest in vitro and in vivo studies [9]. Likewise, *lincRNA*-*p21* can diminish the expression of HIF-1α, reduce the VEGF levels, prevent the cell proliferation and invasion, and finally increase the apoptosis of MHCC97H liver cancer cells [22]. Similarly, the increased *lincRNA*-*p21* expression suppresses invasion, the transition of the cell cycle from G1, cell proliferation, and migration, and also activates the apoptosis of the oesophageal cancer cell line. Actually, it seems that, *lincRNA*-*p21* acts through enhancing the expression of p21 and reduces the expression of cyclin D, and as a result, cell-cycle were arrested [23]. In this context, silencing *lincRNA*-*p21* lead to the reduced levels of p21, rearrangement of chromatin state of some target genes of polycomb, diminish the G1/S efficiency, and enhance the cell proliferation [4]. Another study indicated the enhanced *lincRNA*-*p21* levels and the decreased cell proliferation in mesenchymal stem cells isolated from aged mice compared to younger mice. Notably, the *lincRNA*-*p21* knockdown increased the cell growth via the Wnt/β–catenin signaling pathway [24]. Although *lincRNA*-*p21* may be considered as a tumour suppressor that induce apoptosis, prevent cell growth, and arrest cycle progression, understanding the precise mechanisms of *lincRNA*-*p21* in different processes in carcinogenesis needs further study.

## *LincRNA*-*p21* in tumorigenesis

**Fig. 2:**
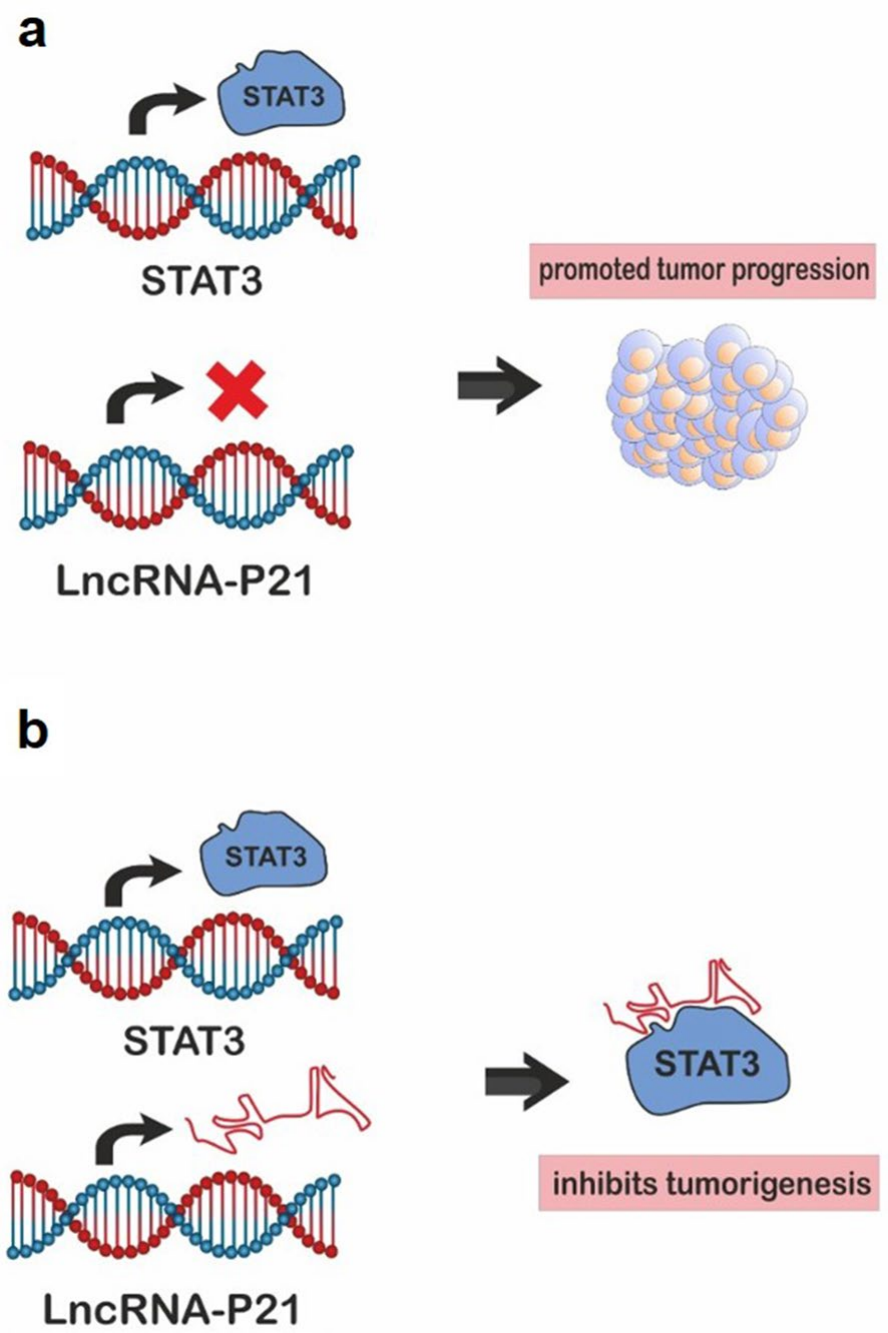
Cross-talk between *lincRNA*-*p21* and STAT3. **a** The reduced expression of *lincRNA*-*p21* inhibits its interaction to STAT3, thus promotes the STAT3 transcriptional activity and induces tumor progression. **b** Overexpression of *lincRNA*-*p21* represses the regulatory activity STAT3 and also inhibits tumorigenesis

Accumulated evidence has been demonstrated that, lncRNAs including *lincRNA*-*p21* play critical roles in tumorigenesis. In fact, *lincRNA*-*p21* inhibits β-catenin translation, leads to the down-regulation of protein levels of β-catenin in HeLa cells [25], which suggest that, it has a potential suppressor of tumorigenesis. However, *lincRNA*-*p21* enhances HIF-1α stability under the hypoxia conditions points that it may also have an oncogenic function and facilitate tumorigenesis [11, 26]. *LincRNA*-*p21* directly binds to STAT3 resulting in the prevention of tumorigenic signals in HNSCC. Briefly, the down-regulation of *lincRNA*-*p21* reduces its interaction with STAT3, and consequently activates the transcriptional activity of STAT3 and promotes tumor progression. On the other hand, *lincRNA*-*p21* up-regulation suppresses the regulatory activity of STAT3, as a result, it inhibits tumorigenesis [9] (Fig. 2). In addition, the low expression of *lincRNA*-*p21* in cancer stem cells led to the activation of tumorigenesis, whereas its increased expression potently repressed the tumorigenesis [27]. Also, Zhang et al. [23] indicated that, *lincRNA*-*p21* inhibits tumorigenesis in esophageal squamous cell carcinoma. Meanwhile, knockdown studies indicated that, endogenous *lincRNA*-*p21* inhibited tumorigenesis and differentiation [28]. Therefore, the lincRNA-p21 has been suggested, as a potent suppressor of tumorigenesis, which could be considered as a valuable therapeutic biomarker in various cancers. For example, the expression of *lincRNA*-*p21* by a novel adenoviral vector in cancer stem cells, could inhibit Wnt/β-catenin signaling pathway resulted in repressing the viability and tumorgenesis of these cells [27]. In addition, Isin et al., reported that, the expression levels of *lincRNA*-*p21* could be a potential diagnostic biomarker in the patients with prostate cancer [29].

## *LincRNA*-*p21* in cancer invasion and metastasis

Tumor invasion and metastasis, which are the symptoms of disease development and the main causes of treatment failure, are known as the main reasons for mortality. Although a number of investigations have assessed the tumor invasion and metastasis, its exact mechanism is not known yet [30]. Previous studies have indicated that, genetic factors including small non-coding RNA and lncRNAs play important roles in the progression of various cancers and malignant metastasis [31, 32].

A number of studies have reported that, lncRNAs including *lincRNA*-*p21* can inhibit the invasion and metastasis of different cancers such as gastric cancer, hepatocellular carcinoma, and colorectal cancer. For example, Chen et al. [12] reported that, in gastric cancer cell lines, cell-to-cell contact was lost and also gained a spindle-like appearance, and vimentin and N-cadherin have enhanced after a *lincRNA*-*p21* knockdown by the Hippo pathway and YAP activation [12].

**Fig. 3:**
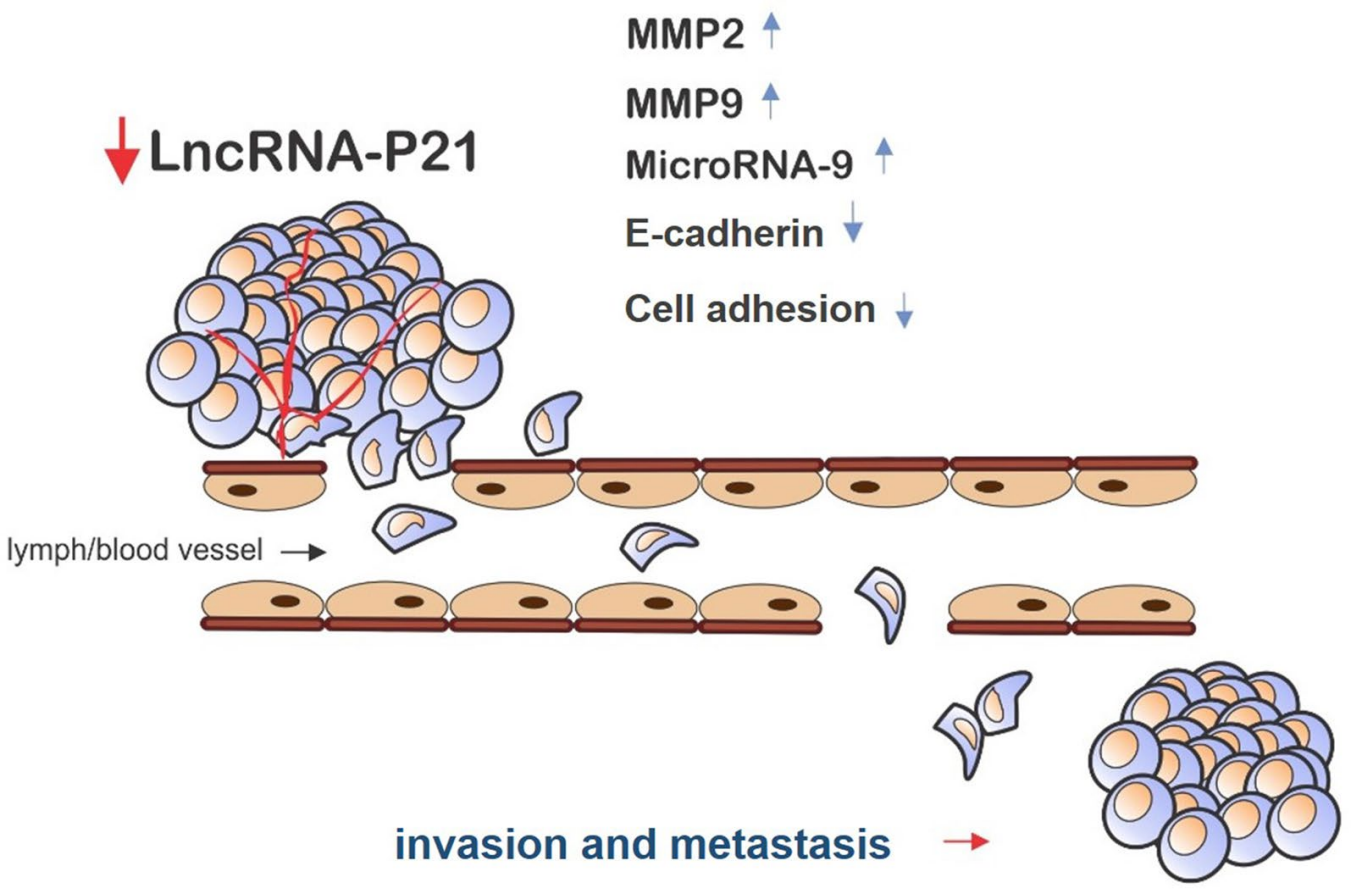
The role of *lincRNA*-*p21* in invasion and metastasis. Down-regulation of *lincRNA*-*p21* leads to enhance MMP2, MMP9, and miRNA-9 expression, as well as reducing the E-cadherin expression and cell adhesion, which finally promotes invasion and metastasis

Moreover, it has been revealed that, *lincRNA*-*p21* activates the epithelial-mesenchymal transition (EMT) by downstream miRNA [33], while Jia et al. [6] have reported that, the increased *lincRNA‐p21* expression can suppress EMT via Notch singling, along with the reduced *lincRNA‐p21* expression that led to a reverse result [6]. In this regard, lncRNA-p21 has been indicated to control the of microRNA-9 expression level negatively. Moreover, microRNA-9 negatively regulates E-cadherin and cell adhesion. Therefore, *lincRNA*-*p21* could repress the development of hepatocellular carcinoma by the miR-9/E-cadherin signaling pathway [33]. Furthermore, high *lincRNA*-*p21* expression reduced the migration and invasion abilities of the head and neck squamous cell carcinoma (HNSCC) cell lines (HN6 and Cal27 cells); however, they have enhanced after the down-regulation of *lincRNA*-*p21*. Correspondingly, the up-regulation of *lincRNA*-*p21* resulted in the decreased matrix metalloproteinase 2 (MMP2) and MMP9 proteins, and vice versa. These resulted proved that, *lincRNA*-*p21* expression suppressed the aggressive manners in these cells [9] (Fig. 3).

## *LincRNA*-*p21* in angiogenesis

Angiogenesis and lymph angiogenesis promoted by chemical factors from tumor cells, are key factors in the growth of tumor and metastasis. Vasculogenesis leads to the formation of the initial vascular network, and consequently differentiate endothelial cells (ECs) from precursors including angioblast. Afterward, angiogenesis activates the formation of the new blood vessels from the existing vessels. In these processes, angiogenic factors such as vascular endothelial growth factor (VEGF) or hypoxia play the key roles [34, 35].

Previous studies have reported that, there are different signaling pathways and new molecules for the induction of angiogenesis. Also, one of the most important novel markers in angiogenesis is lncRNAs [36]. *LincRNA*-*p21* has recently been reported to have critical functions in angiogenesis. For example, in a very interesting study, Castellano et al. [14] found that, *lincRNA*-*p21* play a prognostic role in NSCLC (Non-Small Cell Lung Cancer) via controlling angiogenesis. In this regard, they observed that, the downregulation of *lincRNA*-*p21* under hypoxic conditions causes a reduction in the expression of genes related to angiogenesis (MMP2, PDGFB, VEGFA, FGF2, granulin, and EDIL3). These genes are involved in various processes such as extracellular matrix degradation, metastasis, formation in endothelial cells, angiogenesis, and cell motility. In addition, after silencing *lincRNA*-*p21*, HUVEC tube formation reduced, which suggest a regulatory function of *lincRNA*-*p21* in angiogenesis. Interestingly, the increased levels of *lincRNA*-*p21* in tumor cells from the patients with NSCLC were related to the higher microvascular density [14]. While the inhibition of *lincRNA*-*p21* expression activated angiogenesis in the rats with rheumatoid arthritis (RA). In addition, there was no significant difference in the levels of bFGF, VEGF, and HGF expressions between the RA rats and the RA rats with down-regulating *lincRNA*-*p21* [37]. Also, determining the precise role of *lincRNA*-*p21* in the angiogenesis by in vivo models may provide a promising therapeutic marker.

## *LincRNA*-*P21* in different signaling pathways

**Fig. 4:**
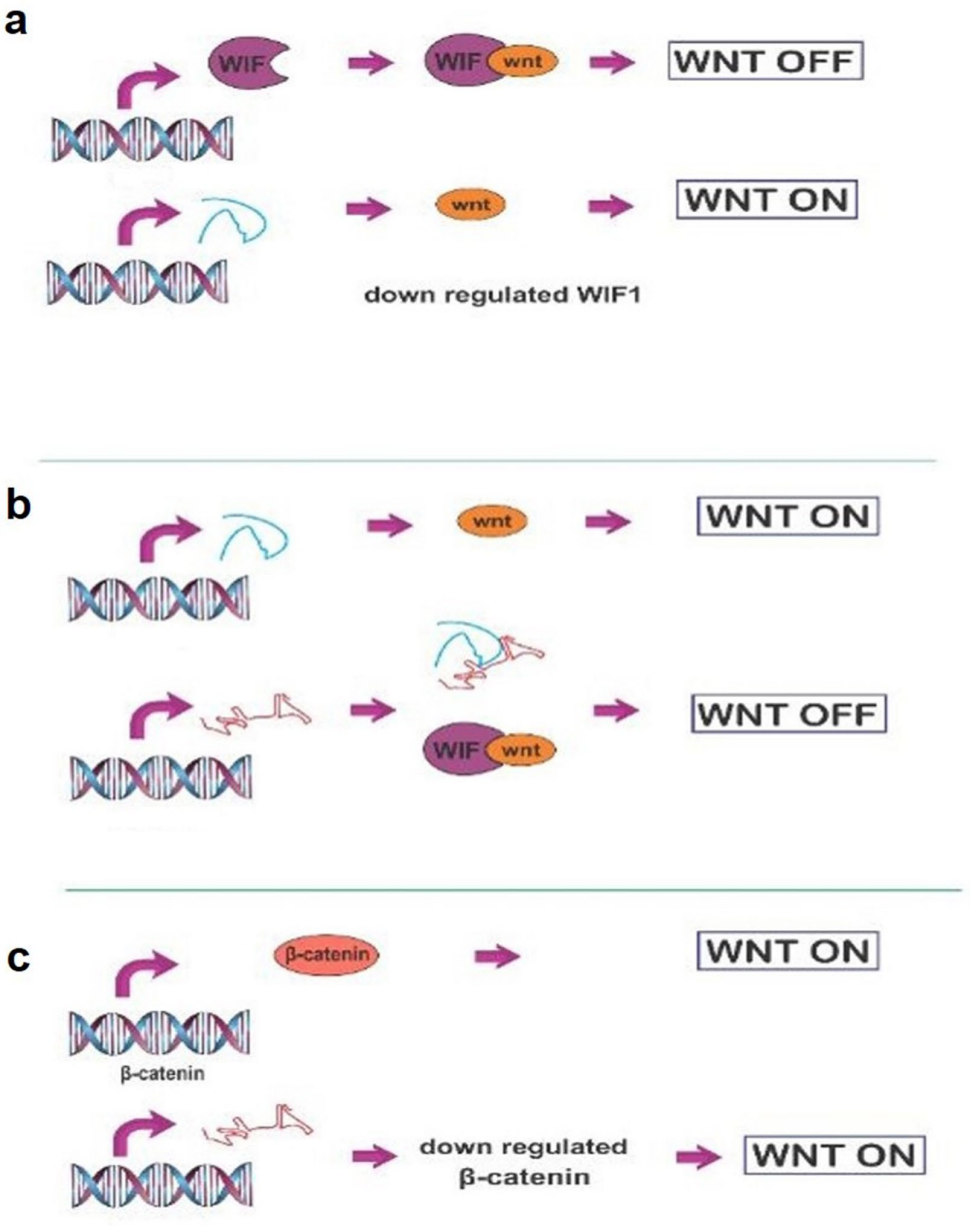
*LincRNA*-*p21* inhibits Wnt/β-catenin pathway. **a** miR-17-5p activates Wnt through down-regulating WIF1. **b***LincRNA*-*p21* suppresses miR-17-5p leading to the inactivation of Wnt. **c***LincRNA*-*p21* down-regulates the β-catenin and inhibits Wnt. WIF1: Wnt inhibitory factor 1

Several studies have evaluated the expression levels of *lincRNA*-*p21* in the different cancer’s tissues and cell lines. Furthermore, numerous studies have revealed the role of this lncRNA in suppressing the cell proliferation and cell growth, as well as the enhancement of apoptosis. Also, it is worth mentioning that, *lincRNA*-*p21* is able to regulate some important signaling pathways in cell proliferation, cell growth, cell cycle, apoptosis, and metastasis including Wnt/β-catenin, miR-9/E-cadherin, Notch signaling, JAK2/STAT3, and AKT/mTOR pathways [6, 9, 33, 38, 39]. In this regard, Wang et al. [39] found that, knockdown of *linc*-*RNA*-*p21* leads to the increased PKM2 expression as well as the activation of AKT or mTOR signaling. PI3K/AKT/mTOR signaling pathway has an important function in cancer progression, such as the prostate cancer [40]. Similarly, the high expression of *lincRNA*-*p21* results in the inhibition of the Wnt/β-catenin pathway, and consequently inactivate the hepatic stellate cells (HSCs) via the impact of miR-17-5p on Wnt inhibitory factor 1 (WIF1) [38] (Fig. 4). In this regard, *lincRNA*-*p21* may enhance the response to radiotherapy for colorectal cancer through the inhibition of the β-catenin signaling pathway and increase in the expression of the Noxa, as a pro-apoptosis gene [41]. Interestingly, the up-regulation of *lincRNA*-*p21* inhibited cell proliferation and the expression of α-SMA and type I collagen in the hepatic stellate cells (HSC). Actually, *lincRNA*-*p21* acts through microRNA-181b to enhance the expressions of PTEN and HSC activation (lincRNA-p21-miR-181b-PTEN Signaling pathway) [42]. Similarly, *lincRNA*-*p21* suppressed the progression of lung cancer cells via the inhibition of cell proliferation and migration as well as the activation of apoptosis in NSCLC. Accordingly, this impact of *lincRNA*-*p21* on lung cancer cells is mediated by the miR-17-5p signaling pathway. Notably, miR-17-5p is a target of *lincRNA*-*p21* [43].

## Crosstalk between *lincRNA*-*p21* and miRNAs

**Fig. 5:**
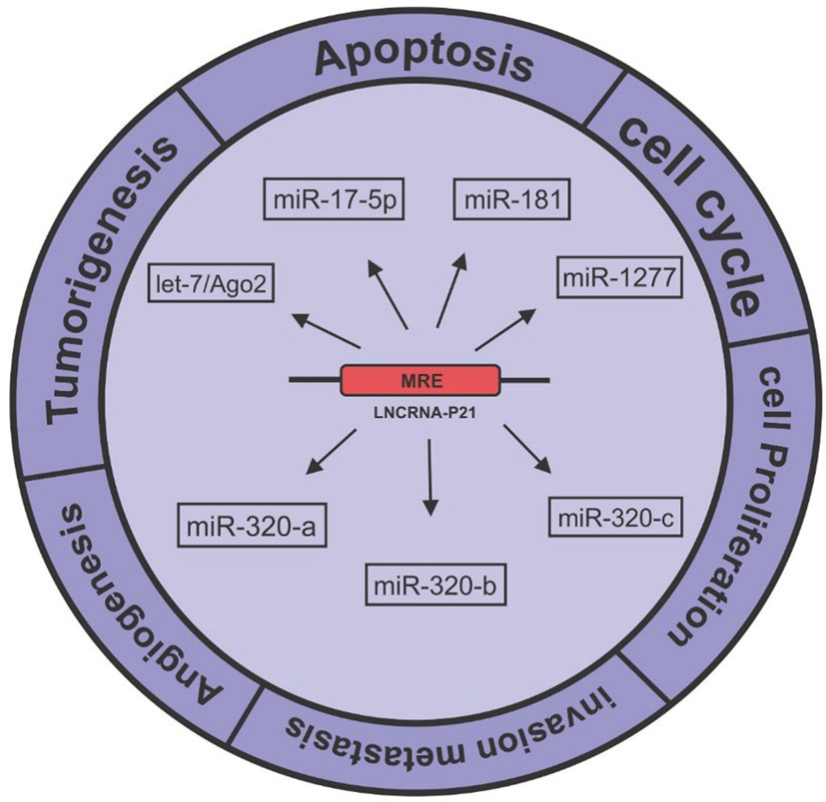
The interplay between *lincRNA*-*p21* and miRNAs for the regulation of various biological processes involved in cancer

lncRNAs have been indicated to have an interaction with other RNAs including miRNAs via nucleic-acid base pairing, and this interaction leads to the lncRNA-mRNA competition for microRNA binding. Based on this fact, lncRNAs are believed to be new competing endogenous RNAs (ceRNAs), which present miRNA binding sites (MREs) [44, 45]. In order for a lncRNA to act as a ceRNA, an MRE in lncRNA requires an incomplete complement to bind to the miRNA. Therefore, the interaction of lncRNAs with miRNAs causes a slow degradation [46]. Novel growing evidence demonstrates that, the interaction between lncRNAs as ceRNAs and miRNAs can regulate various cellular processes and genetic pathways. Also, the disruption of the ceRNAs functions can affect the diverse processes and human diseases including cancer [47]. For instance, Ye et al. [47] by in silico prediction showed that, *lincRNA*-*p21* has a putative MRE for the miR-181 family. They observed that, *lincRNA*-*p21* binds to the miR-181 family and also forms a negative feedback loop with miR-181/PKC-δ that promotes the microglial activation [47]. In line with these results, it was shown that, *lincRNA*-*p21* could inhibit Wnt/β-catenin pathway via binding to miR-17-5p in hepatic stellate cells [38]. Also, an in vitro study displayed the ability of *lincRNA*-*p21* for binding to miR-1277-5p and regulation of the level of miR-1277-5p expression. In addition, the up-regulation of miR-1277-5p inhibited the impact of *lincRNA*-*p21* on the apoptosis and cell viability [48]. Interestingly, miR-320 family (a, b and c) was reported to bind to a 5′ sequence of *lincRNA*-*p21* and regulate its expression through HSF1 (heat-shock factor 1) in MDA-MB-231 cells [49] (Fig. 5).

Mechanisms of the cross-regulation between lncRNAs and miRNAs consisted of four posttranscriptional mechanisms. At first, miRNAs reduced the lncRNAs stabilities, which resulted in the modulation of lncRNAs amount abundance and affected different cellular processes [50]. For example, binding HuR, an RNA-binding protein, to *lincRNA*-*p21* led to the recruitment of let-7/Ago2 to *lincRNA*-*p21*, and the decreased stability of lincRNA-p21. In fact, overexpression of let-7b activated the RNA degradation coding *lincRNA*-*p21* in human cervical carcinoma HeLa cells [25]. Secondly, LncRNAs could sequester miRNAs away from their target mRNAs. Accordingly, these lncRNAs are known as ‘sponges’ or ‘decoys’ for miRNAs, which reduce available miRNAs abundance and increase translations of mRNAs [50]. Interestingly, it has been found that, lncRNA-p21 sponges miR 451 to activate apoptosis in osteoarthritis. In fact, the upregulation of lncRNA-p21 inhibited the miR-451 expression [51]. An in vitro study reported that, LncRNA–p21 elevated the progression of diabetic nephropathy by acting as a sponge for miR-18b. In this study, they found that, miR-18b has reduced by lincRNA-p21 [52]. Thirdly, LncRNAs could compete with miRNAs for binding to their target mRNAs, which consequently removed the regulatory influences of miRNA on mRNAs [50]. Forth, some lncRNAs could generate miRNAs from intronic and exonic region [53]. Altogether, *lincRNA*-*p21* can not only bind to miRNAs directly, as miRNA sponges, and modulate their roles, but it can also be targeted by different miRNAs regulating its expression and function.

## Conclusion and future perspectives

Previous studies support the critical role of *lincRNA*-*p21* in different processes occurring in human cancers. In fact, *lincRNA*-*p21* acts as a tumour suppressor gene inhibiting the cell proliferation, cell survival, tumorigenesis, invasion, metastasis, and angiogenesis. On the other hand, it activates cell apoptosis, in which the expression of several genes happened. It seemed that, *lincRNA*-*p21* may serve as one of the main regulators in various signaling pathways involved in carcinogenesis. It can also involve in response to different therapies for various cancers through regulating the signaling pathways. Moreover, *lincRNA*-*p21* can affect different biological processes by interacting with miRNAs such as miR-181, miR-1277-5p, let-7, and miR-320. Notably, a combination of *lincRNA*-*p21* along with several miRNAs can form a panel of potential biomarkers for various cancers. By indicating several vital roles of *lnicRNAp21* in the pathogenic processes involved in carcinogenesis and its dysregulated expression in human cancers, *lincRNA*-*p21* is suggested as it may serve as a potential diagnostic and prognostic biomarker and a therapeutic target in human cancers. However, these biomarkers, especially in body fluids as a non-invasive way, must be validated using further clinical translation assessments. Meanwhile, the role of this lncRNA in response to radiotherapy suggests that, the regulation of this lincRNA may be a new therapeutic strategy for cancers. However, further clinical studies should be evaluated in the future. In addition, further research conducted on the functions of *lincRNA*-*p21* can reveal the molecular mechanisms of cancer pathogenesis.

## Data Availability

Not applicable.
